# Development and evaluation of improved lines with broad-spectrum resistance to rice blast using nine resistance genes

**DOI:** 10.1186/s12284-019-0292-z

**Published:** 2019-05-06

**Authors:** Haichao Jiang, Zhi Li, Jia Liu, Zhikang Shen, Guanjun Gao, Qinglu Zhang, Yuqing He

**Affiliations:** National Key Laboratory of Crop Genetic Improvement, National Center of Plant Gene Research (Wuhan) and National Center of Crop Molecular Breeding, Huazhong Agricultural University, Wuhan, 430070 China

**Keywords:** Rice, Blast resistance, Marker assisted selection, Gene pyramiding, Natural infection

## Abstract

**Background:**

Rice blast disease is a major restriction in rice production. That is usually managed using chemical pesticides, which are expensive in terms of cost and environment hazards. Use of blast-resistance genes to develop resistant varieties may therefore be a more economical and environmentally friendly method for effective control.

**Results:**

In this study, we improved the blast resistance of four sterile lines, Y58S, GuangZhan63S (GZ63), C815S and HD9802S, by introgression of 9 cloned broad-spectrum blast resistance genes *Pi37*, *Pit*, *Pid3*, *Pigm*, *Pi36*, *Pi5*, *Pi54*, *Pikm* and *Pb1*. Through molecular marker-assisted selection and backcross breeding, 31 single-gene derived lines and 20 double-gene combination lines were obtained. When infected naturally, single-gene lines with *Pigm* or *Pid3* showed significantly enhanced resistance during whole growth period relative to their recurrent parent. Single-gene lines with *Pi37*, *Pi5*, *Pit*, *Pi36*, *Pi54* or *Pikm* showed significantly enhanced resistance in some of the four backgrounds. No obviously enhanced resistance was observed in single-gene line with *Pb1* for the whole growth period. Compared with recurrent parents, most of the double-gene lines showed improved resistance. Among these double-gene lines, lines with *Pi37*/*Pid3*, *Pi5*/*Pi54*, *Pi54*/*Pid3* or *Pigm/Pi37*, exhibited significantly enhanced resistance and observable additive effects.

**Conclusions:**

Two blast resistance genes, *Pigm* and *Pid3*, showed significantly enhanced resistance for the whole rice growth period, and six blast resistance genes *Pi37*, *Pi5*, *Pit*, *Pi36*, *Pi54* or *Pikm* showed significantly enhanced resistance for some of the four backgrounds. Double-gene lines with *Pi37*/*Pid3*, *Pi5*/*Pi54*, *Pi54*/*Pid3* and *Pigm/Pi37* exhibited significantly enhanced resistance and observable additive effects. These lines could be used in rice hybrid and production.

**Electronic supplementary material:**

The online version of this article (10.1186/s12284-019-0292-z) contains supplementary material, which is available to authorized users.

## Introduction

Rice (*Oryza sativa*) is a staple food crop for more than 50% of the world’s population. Rice blast disease is a major restriction on rice production in both tropical and temperate countries, and it is also a major obstacle to hybrid rice production in China due to the relatively narrow genetic base of hybrid rice and the increased use of nitrogen fertilizer (Liu et al. 2010a). The average blast infected area was more than 3.8 million hectares in 1982–1985, with yield losses of several million tons (Sun et al. 1999). In 1993, a yield loss of 1.1 million tons was recorded in Southern China alone. Conventional methods of controlling blast depended on fungicides, which generate additional costs in rice production and chemical contamination of the environment and food. The development and use of resistant varieties with the major resistance genes is therefore one of the most economical and effective ways to control this disease (Koide et al. 2009; Deng et al. 2017).

To date, over 100 blast resistant genes or quantitative trait loci (QTL) have been identified (Su et al. 2015; Vasudevan et al. 2016; Zheng et al. 2016; Xiao et al. 2017). Among them, 35 genes have been cloned (Wang et al. 2017). Many of these resistance genes are clustered on rice chromosomes 6, 11 and 12. Notably, at least 11 resistance genes—including *Pi2*, *Pi9*, *Piz*, *Pizt*, *Pigm*, *Pi22*, *Pi25*, *Pi26*, *Pi40*, *Pi42* and *Pi50*—are concentrated in the short-arm region near the centromere of chromosome 6. Blast resistance gene *Pi37* was mapped from rice cultivar St. No. 1, which encoded a nucleotide-binding site leucine-rich repeat (NBS-LRR) protein on rice chromosome 1 (Chen et al. 2005; Lin et al. 2007). The *Pit* gene was originally identified in cultivar K59 (Hayashi and Yoshida 2009); it was a member of the NBS-LRR family of R genes. The *Pid3* gene was identified by genome-wide comparison of paired NBS-LRR genes and their pseudogene alleles between the two sequenced rice genomes 9311 and Nipponbare, and an allelic *Pid3* in Digu was identified on chromosome 6 (Shang et al. 2009). The broad-spectrum resistance gene *Pigm* was identified from the native Chinese variety Gumei 4. PigmR confers broad-spectrum resistance, whereas PigmS competitively attenuates PigmR homodimerization to suppress resistance. The expression of PigmS that triggered PigmR-mediated resistance is subjected to tight epigenetic regulation (Deng et al. 2009; Deng et al. 2017). *Pi36* identified in an *indica* cultivar, Q61, was mapped on chromosome 8 and encoded as an NBS-LRR protein (Liu et al. 2005; Liu et al. 2010b). The *Pi5* gene was identified from RIL260, and the resistance of *Pi5* to *Magnaporthe oryzae* requires the presence of the two coiled-coil NBS-LRR genes *Pi5–1* and *Pi5–2* (Lee et al. 2009). *Pi54* was originally identified from the rice variety Tetep and was mapped on chromosome 11 with two tightly linked simple sequence repeat (SSR) markers TRS26, TRS33 and a functional marker *Pi54*-MAS (Sharma et al. 2005; Ramkumar et al. 2011). *Pi54* was ∼2.5 Mb away from the *Pik* locus on rice chromosome 11 (Sharma et al. 2010). It had been reported that the *Pik* locus was actually a cluster of genes including *Pikm*, *Pik-h* and *Pik-p* presenting on rice chromosome 11 (Ashikawa et al. 2008; Zhai et al. 2014; Yuan et al. 2011). *Pikm*-specific rice blast resistance is conferred by a combination of two genes, *Pikm1-TS* and *Pikm2-TS*, with an NBS-LRR (Ashikawa et al. 2010). The *Pb1* gene derived from an *indica* cultivar, Modan, and is characterized by durability of resistance and adult/panicle blast resistance (Hayashi et al. 2010; Table 1).

**Table 1:** List of blast resistance genes using in this study

Gene	Chromosome	Encoding protein	Donor	Reference
*Pi37*	1	NBS-LRR	St. No. 1	Lin et al. 2007
*Pit*	1	NBS-LRR	K59	Hayashi and Yoshida 2009
*Pid3*	6	NBS-LRR	Digu	Shang et al. 2009
*Pigm*	6	NBS-LRR	Gumei4	Deng et al. 2009
*Pi36*	8	NBS-LRR	Q61	Liu et al. 2005
*Pi5*	9	NBS-LRR	IRL260	Lee et al. 2009
*Pi54*	11	NBS-LRR	Tetep	Sharma et al. 2005
*Pikm*	11	NBS-LRR	Tsuyuake	Ashikawa et al. 2008
*Pb1*	11	NBS-LRR	Modan	Hayashi et al. 2010

Marker-assisted selection (MAS) is a highly efficient breeding approach that could offer an opportunity to select the targeted gene rapidly and precisely (Tanksley et al. 1989). It is a promising method to provide broad-spectrum and durable rice blast resistance through gene or QTL pyramiding (Tabien et al. 2002). Recently, the development of near-isogenic lines or the pyramiding of different resistance genes has been applied in blast resistance breeding programs by marker-assisted selection. Three blast resistance genes (*Pi1*, *Pi2* and *D12*) were introduced into rice variety Jin23B (Jiang et al. 2012), and *Pi9*, *Pizt* and *Pi54* were recently introduced into rice variety 07GY31 by marker assisted backcross breeding (Xiao et al. 2017). Evaluation of blast resistance suggested that single or polygene pyramid lines showed significantly enhanced resistance relative to control.

Y58S, GZ63S, C815S and HD9802S are elite rice varieties in rice production in China, but these four varieties and their derived hybrids are highly susceptible to blast. Improvement of blast resistance in Y58S, GZ63S, C815S and HD9802S is therefore critical in utilizing the hybrids in rice production in China.

In this study, 9 cloned blast resistance genes, *Pi37*, *Pit*, *Pid3*, *Pigm*, *Pi36*, *Pi5*, *Pi54*, *Pikm* and *Pb1*, were introgressed into male sterile lines including Y58S, GZ63S, C815S and HD9802S. Our objective was to evaluate the natural resistance performance of these cloned blast resistance genes and to improve hybrid rice blast resistance in production.

## Materials and methods

### Plant materials

Nine rice varieties, Q1333 (*Pi37*), K59 (*Pit*), Digu (*Pid3*), Gumei4 (*Pigm*), Q61 (*Pi36*), RIL260 (*Pi5*), Tsuyuake (*Pikm*), Tetep (*Pi54*) and Modan (*Pb1*), were used as donors of the cloned resistance genes (Table 1). Four male sterile rice lines (Y58S, GZ63S, C815S and HD9802S), which are the main female parent of *indica* hybrid rice in southern China, were used as the recurrent parents. The F_1_, BC_1_F_1_, BC_2_F_1_ and BC_3_F_1_ lines were derived from crosses between the recurrent parents and the donor parents. The BC_1_F_2_ and BC_2_F_2_ populations were developed from the BC_1_F_1_ and BC_2_F_1_ resistant individuals. These populations were used for genetic and phenotypic analysis. The susceptible variety CO39 was used as a negative control.

### DNA extraction and genotyping

For MAS during each generation, DNA was isolated from the leaf tissues of the parent, BC_n_F_1_, BC_n_F_2_ and F_2_ plants using the CTAB method. In the MAS system, *Pit* was detected using SSR marker RM10125 and InDel marker RMLTJ-3; *Pi37* was detected using SSR marker RM11726 and InDel marker RMLJ-1; and *Pigm* was detected using InDel marker Pi2–4 and SSR markers HC26 and HC3. The flanking InDel marker RML3J-1 and SSR marker RM19951 were used to detected *Pid3*; the SSR marker RM22385 and InDel marker RMLJ-2 were used to detected *Pi36*; and two SSR markers, RM24019 and RM24034, and one InDel marker, RMLJ-7, were used to detected *Pi5*. Three tightly linked SSR markers RM27150, RM27181 and RM27189 were used to confirm gene *Pi54*. Two tightly linked SSR markers RM26998 and RM26964 were used to confirm *Pb1*. SSR marker RM224 and InDel marker RMLMJ-1 were used to confirm *Pikm* (Additional file 1: Table S1). The InDel markers RMLTJ-3, RMLJ-1, Pi2–4, RML3J-1, RMLJ-2, RMLJ-7 and RMLMJ-1 were designed based on sequence alignments of the two genome references of Nipponbare and 93–11 (Additional file 1: Table S1). The SSR analysis was carried out essentially according to the procedures described by Wu and Tanksley (1993).

### Crossing and selection scheme

**Fig. 1:**
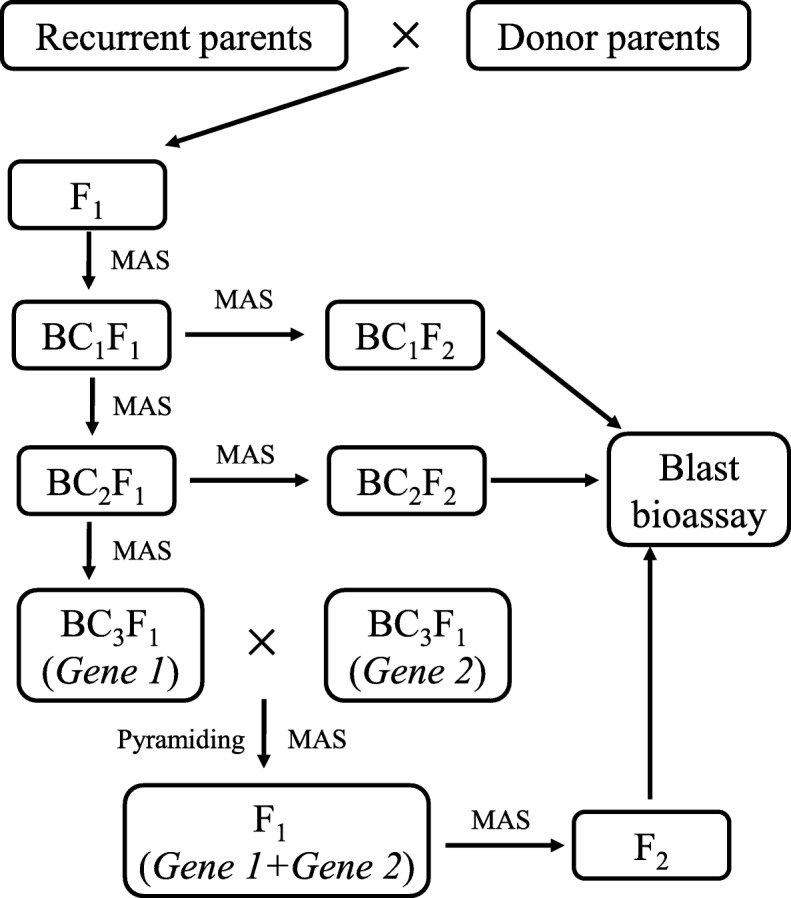
Strategy to develop improved populations and resistance identification

**Fig. 2:**
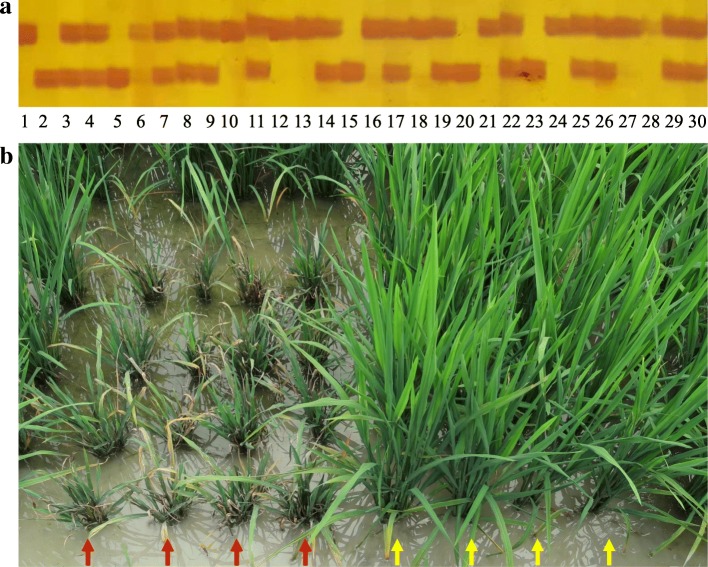
Identification of improved blast resistant lines with MAS. **a** PCR amplification of the marker LJ3–1 for blast resistance gene *Pid3* in the BC_2_F_2_ population with the background of GZ63S. Lines 1, 2 and 3 are Digu (resistance homozygous), GZ63S (infected homozygous) and heterozygous (Digu/GZ63S), respectively. **b** Red arrows indicate susceptible parents GZ63S, yellow arrows show the resistant lines carrying target gene *Pid3*

Q1333 (*Pi37*), K59 (*Pit*), Digu (*Pid3*), Gumei4 (*Pigm*), Q61 (*Pi36*), RIL260 (*Pi5*), Tsuyuake (*Pikm*), Tetep (*Pi54*) and Modan (*Pb1*) were crossed separately with Y58S, GZ63S, C815S and HD9802S, and their F_1_ hybrids were backcrossed with Y58S, GZ63S, C815S and HD9802S to obtain the BC_1_F_1_ populations (Fig. 1). Markers closely linked with the blast resistance genes were used to check the target genes among the above BC_1_F_1_ populations (Fig. 1; Fig. 2a). Twenty plants with the target genes from each BC_1_F_1_ population were selected to backcross with the corresponding parents up to BC_3_F_1_. From each generation, plants carrying single gene *Pi37*, *Pit*, *Pid3*, *Pigm*, *Pi36*, *Pi5*, *Pi54*, *Pikm* and *Pb1* in the background of Y58S, GZ63S, C815S and HD9802S were obtained. After selfing, the BC_1_F_2_ and BC_2_F_2_ populations were obtained, which were then used to evaluate the effects of individual genes in different backgrounds. In the BC_3_F_1_ populations, two genes in the same background were crossed with each other, and then the F_1_ hybrids and the corresponding F_2_ population were obtained (Fig. 1).

### Scoring rice blast

The BC_1_F_2_, BC_2_F_2_ and F_2_ families of the blast resistance genes *Pi37*, *Pit*, *Pid3*, *Pigm*, *Pi36*, *Pi5*, *Pi54*, *Pikm* and *Pb1* in the background of Y58S, GZ63S, C815S and HD9802S were planted in a randomized complete block design in 2014, 2015 and 2016 in Xianfeng, Hubei Province, China. Xianfeng is a mountainous area at an altitude of 600 m, with an average temperature of approximately 25 °C, showing high humidity and heavy fog annually. The tests were performed in three replications. In each replication, each of the plots consisted of 8 rows with 12 plants per row at a planting density of 16 cm between plants and 26.5 cm between rows. To adequately induce blast disease infection, the diseased straws collected the previous year were sown evenly in each plot and the highly susceptible variety, CO39, was planted at both sides of each row and around the population. Field management essentially followed normal agricultural practices with the exception of not using bactericides.

All the plants were scored for leaf blast at the tillering stage and were recorded for neck blast severity at maturity. The most seriously infected leaf among the top two or three new leaves was scored for each plant at the tillering stage, as determined using the 0–9 scale rating system from IRRI (2002). Neck blast severity was recorded as a percentage of the infection on the neck of the rice panicle at physiological maturity. The number of panicles showing symptoms of neck blast was expressed as percent infection.

### Statistical analysis

The data obtained from the experiments were statistically analyzed using analysis of variance (ANOVA) of respective experimental designs. Phenotypic and genotypic data were collected for each individual plant in the BC_1_F_2_ and BC_2_F_2_ populations. The additive effect (A), dominant effect (D) and phenotypic variation explained (PVE) of the resistance genes were analyzed in a segregated population between different genotypes at *P* = 0.05 and 0.01 significance. The analyses of these statistical parameters were carried out using the statistical software SPSS 20.0.

## Results

### Improved blast resistance materials obtained through MAS

Seven blast resistance genes, *Pi37*, *Pit*, *Pid3*, *Pigm*, *Pi36*, *Pi5*, *Pi54*, were introgressed into Y58S, GZ63S, C815S and HD9802S separately following a recurrent backcrossing procedure, combined with MAS as described in Fig. 1. In addition, *Pikm* for blast resistance was introgressed into C815S and HD9802S, and *Pb1* for blast resistance was introgressed into GZ63S, using the marker-assisted backcross breeding method (Fig. 1). Thirty-one BC_1_F_2_, BC_2_F_2_ and BC_3_F_1_ lines with single resistance genes were obtained.

The improved lines containing a single resistance gene in BC_3_F_1_ progenies were intercrossed with each other to pyramid two resistance genes. The F_1_ plants containing two resistance genes were selected through the linked DNA markers of each target gene. Two gene pyramiding lines were also obtained, including Y58S (*Pi37*/*Pi54*), Y58S (*Pi5*/*Pi37*), Y58S (*Pi5*/*Pigm*), Y58S (*Pigm*/*Pi54*), Y58S (*Pi37*/*Pid3*), Y58S (*Pi37*/*Pigm*) in Y58S background, GZ63S (*Pit*/*Pigm*), GZ63S (*Pit*/*Pb1*), GZ63S (*Pi5*/*Pit*), GZ63S (*Pi5*/*Pi37*), GZ63S (*Pi36*/*Pb1*), GZ63S (*Pi5*/*Pigm*), GZ63S (*Pi36*/*Pid3*), GZ63S (*Pb1*/*Pigm*), GZ63S (*Pi5*/*Pi54*), GZ63S (*Pid3*/*Pi54*) in GZ63S background, C815S (*Pit*/*Pigm*), C815S (*Pi5*/*Pit*), C815S (*Pi5*/*Pigm*) and C815S (*Pi37*/*Pigm*) in C815S background (Fig. 1).

### Blast resistance of the donor parents and recurrent parents

Four recurrent parents and nine donor parents were planted in Xianfeng in 2014, 2015 and 2016. Each variety was planted in three replications with 12 plants per plot. The resistance scores for leaf blast for Y58S were 6.99, 6.57 and 6.53, and the percentages of infection for neck blast were 98.71%, 95.28% and 100% in 2014, 2015 and 2016, respectively. The resistance scores for leaf blast for GZ63 were 7.42, 7.75 and 7.02 and the percentages of infection for neck blast were 98.83%, 100% and 100% in 2014, 2015 and 2016, respectively. At the disease nursery of Xianfeng, the resistance scores for leaf blast for C815S and HD9802S were 6.90, 6.38 and 6.98 and 6.50, 6.68 and 6.45, and the percentages of infection for neck blast were 99.08%, 93.15% and 100% and 98.17%, 100% and 100%, in 2014, 2015 and 2016, respectively. Four recurrent parents, Y58S, GZ63S, C815S and HD9802S, showed medium or high susceptibility to leaf blast at the tillering stage and high susceptibility to neck blast at maturation in Xianfeng in 2014, 2015 and 2016 (Table 2). These results illustrated a serious loss of blast resistance in the main hybrid rice, and it is imperative to improve the blast resistance in hybrid rice in China.

**Table 2:** Phenotypic average value of recurrent parents and donor parents in 2014, 2015 and 2016

		Recurrent parents				Donor parents								
		Y58S	GZ63S	C815S	HD9802S	K59 (*Pit*)	Tsuyuake (*Pikm*)	Modan (*Pb1*)	RIL260 (*Pi5*)	Q61 (*Pi36*)	Q1333 (*Pi37*)	Gumei4 (*Pigm*)	Digu (*Pid3*)	Tetep (*Pi54*)
2014	LB	6.99	7.42	6.90	6.50	5.70	4.40	4.60	5.70	8.50	3.80	2.98	3.10	3.02
	NB(%)	98.71	98.83	99.08	98.17	97.93	98.85	98.93	97.10	100.00	21.11	3.00	3.04	2.05
2015	LB	6.57	7.75	6.38	6.68	2.33	2.67	3.30	1.75	7.67	3.01	1.33	3.08	1.00
	NB(%)	95.28	100.00	93.15	100.00	100.00	100.00	100.00	79.08	40.17	35.95	36.99	7.97	1.00
2016	LB	6.53	7.02	6.98	6.45	3.92	4.08	3.17	4.83	6.75	1.99	1.92	1.17	2.02
	NB(%)	100.00	100.00	100.00	100.00	100.00	100.00	100.00	78.06	62.03	5.99	5.04	7.97	1.00

*LB* Leaf blast, *NB* Neck blast

The resistance scores for leaf blast at the tillering stage and the percentage of infection for neck blast at maturation were 5.70 and 97.93% for K59; 4.40 and 98.85% for Tsuyuake; 4.60 and 98.93% for Modan; 5.70 and 97.10% for RIL260; 8.50 and 100.00% for Q61; 3.80 and 21.11% for Q1333; 2.98 and 3.00% for Gumei4; 3.10 and 3.04% for Digu; 3.02 and 2.05% for Tetep, respectively, in 2014 (Table 2). The resistance scores for leaf blast at tillering stage for these nigh varieties were 2.33, 2.67, 3.30, 1.75, 7.67, 3.01, 1.33, 3.08 and 1.00, respectively, in 2015, and 3.92, 4.08, 3.17, 4.83, 6.75, 1.99, 1.92, 1.17 and 2.02, respectively, in 2016 (Table 2). The percentages of infection for neck blast at maturity stage for the nine variety were 100.00%, 100.00%, 100.00%, 79.08%, 40.17%, 35.95%, 36.99%, 7.97% and 1.00%, respectively in 2015 and 100.00%, 100.00%, 100.00%, 78.06%, 62.03%, 5.99%, 5.04%, 7.97% and 1.00%, respectively in 2016 (Table 2). These results showed that Gumei4, Digu, Tetep and Q1333 had high resistance to leaf blast and neck blast during these 3 years. K59, Tsuyuake, Modan and RIL260 had high resistance to leaf blast in 2015 and medium resistance to leaf blast in 2014 and 2016, but were susceptible to neck blast for all 3 years. The resistance scores of Q61 to leaf blast and percentage of infection for neck blast showed that the variety was susceptible to both leaf blast and neck blast.

### Blast resistance of the genes in different backgrounds

Seven blast resistance genes (*Pi37*, *Pit*, *Pid3*, *Pigm*, *Pi36*, *Pi5*, *Pi54*) were introduced into four recurrent parents Y58S, GZ63S, C815S and HD9802S, one blast resistance gene (*Pikm*) was introduced into C815S and HD9802S, and one blast resistance gene (*Pb1*) was introduced into GZ63S. The resistance to leaf blast and neck blast of single gene introduced lines was tested in 2014 and 2015 in Xianfeng. *Pid3* showed greater resistance to leaf blast and neck blast in the background of Y58S, GZ63S, C815S and HD9802S than the control in 2014 and 2015 (Table 3; Fig. 2). *Pigm* showed significantly enhanced resistance to leaf blast in the background of Y58S, C815S and HD9802S in 2015, and it showed significantly enhanced resistance to neck blast in the background of Y58S, GZ63S, C815S and HD9802S in 2014 and 2015 (Table 3). *Pi5* showed significantly enhanced resistance to leaf blast in the background of C815S in 2014 and in the background of GZ63S and HD9802S in 2015 and significantly enhanced resistance to neck blast in the background of GZ63S in 2015 (Table 3). *Pi37* showed significantly enhanced resistance to leaf blast in the background of GZ63S in 2014 and in the background of Y58S and C815S in 2015 and significantly enhanced resistance to neck blast in the background of Y58S and GZ63S in 2015. *Pi54* showed significantly enhanced resistance to leaf and neck blast in the background of Y58S, GZ63S and HD9802S in 2015. Except for *Pb1*, all of the blast resistance genes showed enhanced resistance to leaf or neck blast in at least one background from a recurrent parent. However, the blast resistance of *Pi5*, *Pi36* and *Pit* was not stable in most recurrent parents (Table 3).

**Table 3:** Broad-spectrum resistance of different resistance genes over whole growth period in 2014 and 2015 in Xianfeng

Gene	Recurrent parent	Leaf Blast						Neck Blast					
		2014			2015			2014			2015		
		A	D	PVE (%)	A	D	PVE (%)	A	D	PVE (%)	A	D	PVE (%)
*Pi5*	Y58S	− 0.05	− 0.10	0.41	−0.02	0.09	0.94	–	–	–	0.00	0.00	0.00
	GZ63S	0.00	0.00	0.00	− 0.72	0.15	13.81^a^	0.00	0.00	0.00	− 0.13	− 0.14	10.38^a^
	C815S	−0.95	− 0.82	39.69^b^	/	/	/	–	–	–	/	/	/
	HD9802S	−0.26	− 0.07	3.84	− 0.69	− 0.45	20.78^b^	–	–	–	− 0.05	− 0.14	2.19
*Pi36*	Y58S	−0.03	− 0.11	0.55	− 0.27	0.07	4.32	0.00	0.00	0.00	−0.03	−0.04	0.11
	GZ63S	0.04	−0.68	2.48	−0.23	0.15	8.35	0.00	0.00	0.00	0.00	0.00	0.00
	C815S	0.25	−0.08	4.40	− 0.33	0.10	3.76	0.00	0.00	0.00	−0.02	0.02	5.81^a^
	HD9802S	−0.33	0.18	5.75	0.10	−0.10	0.08	0.00	0.00	0.00	−0.04	0.01	0.54
*Pi37*	Y58S	−0.14	−0.10	3.10	−0.58	− 0.04	7.17^a^	− 0.19	−0.10	9.73^a^	−0.07	− 0.06	6.06^a^
	GZ63S	−0.61	−0.67	13.50^b^	/	/	/	−0.36	−0.44	59.85^b^	/	/	/
	C815S	0.00	0.00	0.00	−0.88	0.74	21.06^b^	–	–	–	−0.05	0.06	0.74
	HD9802S	/	/	/	−0.04	−0.16	0.18	/	/	/	0.00	0.00	0.00
*Pigm*	Y58S	−1.46	−1.09	30.54^b^	−0.64	−0.96	9.25^a^	−0.40	−0.36	46.44^b^	−0.39	− 0.23	41.04^b^
	GZ63S	/	/	/	−0.18	−0.05	1.53	/	/	/	−0.30	−0.33	26.00^b^
	C815S	−0.12	0.02	0.84	−0.48	−0.06	6.47^a^	−0.17	− 0.08	7.88^a^	− 0.28	−0.18	33.00^b^
	HD9802S	−0.11	0.05	0.75	−1.75	− 1.20	42.84^b^	–	–	–	−0.38	−0.30	39.82^b^
*Pi54*	Y58S	−0.27	−0.13	6.18^a^	−0.83	− 0.39	23.09^b^	− 0.18	−0.19	15.81^b^	−0.30	0.16	30.13^b^
	GZ63S	0.14	0.27	2.23	−0.60	−0.13	20.32^b^	0.01	−0.05	0.35	−0.13	−0.03	12.17^a^
	C815S	0.21	−0.07	2.91	/	/	/	–	–	–	/	/	/
	HD9802S	−0.08	0.01	0.28	−0.44	−0.43	6.38^a^	–	–	–	−0.28	− 0.03	30.88^b^
*Pid3*	Y58S	−0.90	−0.79	31.99^b^	−1.23	0.42	17.76^b^	−0.34	−0.31	40.77^b^	−0.38	− 0.22	64.61^b^
	GZ63S	−1.09	−0.69	32.62^b^	−1.66	−1.53	75.48^b^	−0.39	−0.22	39.26^b^	−0.38	− 0.35	64.38^b^
	C815S	−1.76	−1.60	48.83^b^	−0.69	−0.51	17.88^a^	−0.35	− 0.05	25.65^b^	− 0.19	0.06	23.42^b^
	HD9802S	−0.67	−0.58	36.41^b^	−0.90	0.10	34.49^b^	−0.30	−0.01	35.19^b^	−0.32	0.11	32.42^b^
*Pit*	Y58S	−0.09	−0.41	3.23	−0.18	− 0.50	4.22	0.00	0.00	0.00	0.00	0.00	0.00
	GZ63S	−0.13	−0.23	2.26	−0.69	0.47	9.74^a^	0.00	0.00	0.00	−0.01	0.00	7.71
	C815S	0.18	0.08	3.14	−0.14	−0.03	1.78	0.00	0.00	0.00	−0.05	0.03	1.82
	HD9802S	/	/	/	−0.54	−0.51	13.03^a^	/	/	/	−0.05	0.03	5.18
*Pb1*	GZ63S	/	/	/	−0.02	−0.36	0.12	/	/	/	0.00	0.00	0.00
*Pikm*	C815S	−1.61	−1.18	53.44^b^	/	/	/	0.00	0.00	0.00	/	/	/
	HD9802S	−1.05	−1.04	40.33^b^	/	/	/	0.00	0.00	0.00	/	/	/

*A* Additive effect, *D* Dominant effect, *PVE (%)* Phenotypic variation explained (%); ^a^ and ^b^ denote significance at the 0.05 and 0.01 probability levels, respectively. “**-**” indicates no date were collected, “**/**” indicates no materials under the background

### Blast resistance of two gene pyramiding lines

**Fig. 3:**
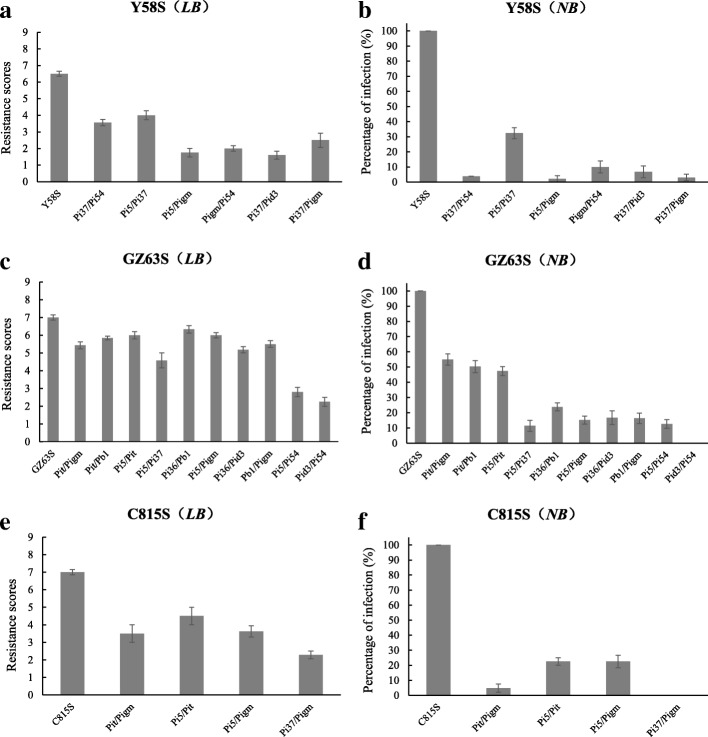
Blast resistance scores of homozygous plants of pyramiding populations under different backgrounds. Shown are the average values for leaf blast (LB) in different pyramiding populations in the backgrounds Y58S (**a**) GZ63S (**c**) and C815S (**e**) and the average value of neck blast (NB) of different pyramiding populations in the backgrounds Y58S (**b**), GZ63S (**d**) and C815S (**f**)

Through MAS, 6, 10 and 4 lines with two-gene pyramiding in Y58S, GZ63S and C815S backgrounds were obtained. The blast resistance of two-gene pyramiding lines was scored for leaf blast at the tillering stage and neck blast at maturation in 2016. The resistance scores for leaf blast were 3.56 for Y58S (*Pi37*/*Pi54*), 4.00 for Y58S (*Pi5*/*Pi37*), 1.75 for Y58S (*Pi5*/*Pigm*), 2.00 for Y58S (*Pigm*/*Pi54*), 1.60 for Y58S (*Pi37*/*Pid3*), and 2.50 for Y58S (*Pi37*/*Pigm*), while the percentage of infection for neck blast was 3.88%, 32.34%, 2.08%, 10.00%, 6.80% and 3.05%, respectively (Fig. 3). At the tillering stage, the resistance scores for leaf blast for pyramiding lines GZ63S (*Pit*/*Pigm*), GZ63S (*Pit*/*Pb1*), GZ63S (*Pi5*/*Pit*), GZ63S (*Pi5*/*Pi37*), GZ63S (*Pi36*/*Pb1*), GZ63S (*Pi5*/*Pigm*), GZ63S (*Pi36*/*Pid3*), GZ63S (*Pb1*/*Pigm*), GZ63S (*Pi5*/*Pi54*), GZ63S (*Pid3*/*Pi54*) were 5.42, 5.84, 6.00, 4.58, 6.33, 6.00, 5.18, 5.50, 2.80 and 2.25, respectively. At maturation, the percentage of infection for neck blast was 55.02%, 50.29%, 47.30%, 11.40%, 23.83%, 15.20%, 16.69%, 16.34%, 12.67% and 0%, respectively for the 10 pyramiding lines in GZ63S background (Fig. 3). The resistance scores for the two-gene pyramiding lines C815S (*Pit*/*Pigm*), C815S (*Pi5*/*Pit*), C815S (*Pi5*/*Pigm*), C815S (*Pi37*/*Pigm*) were 3.50, 4.50, 3.62 and 2.29, respectively, for leaf blast and 4.76%, 22.50%, 22.50% and 0%, respectively, for neck blast (Fig. 3). By contrast, the resistance scores of the recurrent parents Y58S, GZ63S and C815S were 6.53, 7.02 and 6.98, respectively, for leaf blast and the percentage of infection for neck blast was 100.00%, 100.00% and 100.00%, respectively. These results indicate that the pyramiding lines were more strongly resistant to blast than the control.

**Fig. 4:**
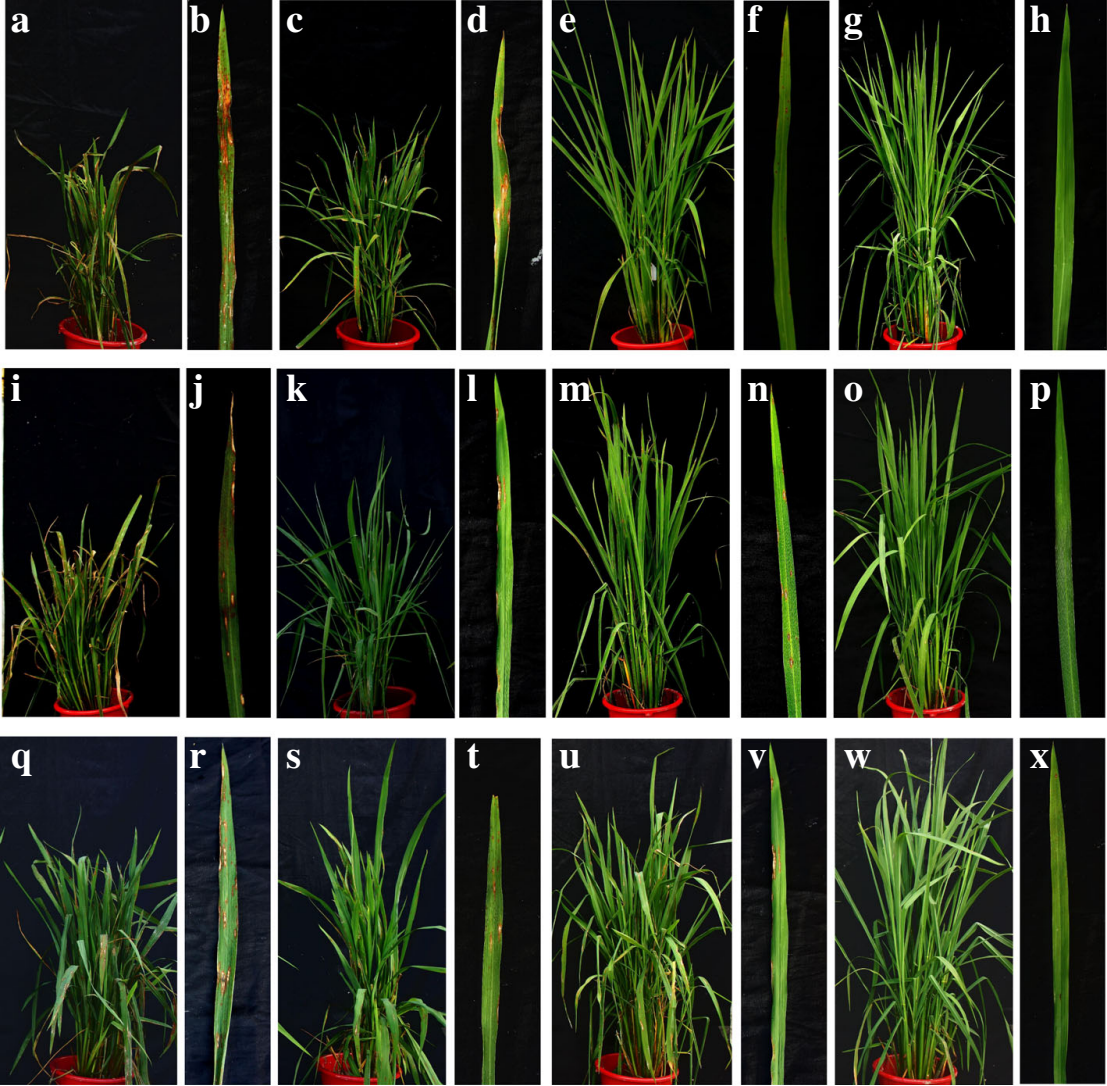
Phonotypes of improved lines and controls for leaf blast under natural infection conditions. **a**, **b** Phenotype of recurrent parent Y58S for leaf blast under natural infection conditions. Phenotypes of improved plants carrying resistance gene under Y58S background to leaf blast under natural infection condition: **c**, **d** Y58S (*Pi54*); **e**, **f** Y58S (*Pid3*); **g**, **h** Y58S (*Pi54 + Pid3*). **i**, **j** phenotype of recurrent parent GZ63S to leaf blast under natural infection condition. Phenotype of improved plants carrying resistance gene under GZ63S background to leaf blast under natural infection condition; **k**, **l** GZ63S (*Pi54*); **m**, **n** GZ63S (*Pi5*); **o**, **p** GZ63S (*Pi54 + Pid3*). **q**, **r** phenotype of recurrent parent C815SS to leaf blast under natural infection condition. Phenotype of improved plants carrying resistance gene under C815SS background to leaf blast under natural infection condition. **s**, **t** C815SS (*Pigm*); **u**, **v** C815SS (*Pi37*); **w**, **x** C815SS (*Pigm + Pi37*)

In the background of Y58S, the leaf blast sores for *Pi37*, *Pid3* and *Pi37*/*Pid3* were 3.90, 1.83 and 1.60, respectively, which explained the phenotype variation of 13.89%, 11.39% and 24.46%, respectively. The percentage of infection for neck blast for *Pi37*, *Pid3* and *Pi37*/*Pid3* was 12.98%, 5.15% and 3.05%, respectively, which explained the phenotype variation of 11.91%, 16.79% and 32.38% (Table 4; Fig. 4). In the background of Y58S, the two-gene pyramiding line *Pi37*/*Pid3* showed enhanced resistance to blast compared with single gene lines for leaf and neck blast. In the background of GZ63S, the resistance to leaf blast of the two-gene pyramiding lines of *Pi5*/*Pi54* and *Pid3*/*Pi54* were 2.80 and 2.25, while the resistance to neck blast was 12.67% and 0%, which showed significant difference from the single gene lines (Table 4; Fig. 3; Fig. 4). In the background of C815S, the leaf and neck blast of two-gene pyramiding line *Pi37*/*Pigm* were 2.29 and 0%, which showed significant difference from the single gene lines (Table 4; Fig. 3). From the results above, all of the pyramiding lines showed greater resistance to blast than the single gene lines, especially for neck blast.

**Table 4:** broad-spectrum resistance of resistance genes over whole growth period in 2016 in Xianfeng

Recurrent parent	Gene	Leaf blast		Neck blast	
		Phenotypic value	PVE (%)	Phenotypic value (%)	PVE (%)
Y58S	None	6.53	0	100.00	0
	*Pid3*	1.83	11.39*	5.15	16.79**
	*Pi37*	3.90	13.89*	12.98	11.91*
	*Pi54*	3.96	12.30*	3.76	31.26**
	*Pi37 + Pid3*	1.60	24.46**	3.05	32.38**
	*Pi37 + Pi54*	3.56	14.48*	3.88	30.28**
GZ63S	None	7.02	0	100.00	0
	*Pi5*	3.60	12.84*	16.49	9.53*
	*Pi54*	3.73	9.52*	15.67	12.90**
	*Pid3*	3.07	19.95**	2.50	48.10**
	*Pi5 + Pi54*	2.80	19.40**	12.67	17.39**
	*Pi54 + Pid3*	2.25	22.74**	0.00	55.80**
C815S	None	6.98	0	100.00	0
	*Pigm*	3.02	10.12*	6.86	9.27*
	*Pi37*	2.95	22.64**	1.82	13.55**
	*Pigm + Pi37*	2.29	24.47**	0.00	46.37**

PVE (%), phenotypic variation explained (%); Significantly different from BC lines carrying blast resistance genes and corresponding recurrent parents lacking these genes at **P < 0.01 and *P < 0.05

## Discussion

Since the 1980s, several blast resistance genes have been identified and transferred into elite susceptible varieties, producing a series of improved cultivars with blast resistance. In recent years, MAS has been employed for transferring blast resistance genes to new varieties. Among the 35 cloned genes, the breeding application of blast genes *Pi1* and *Pi2* has often been reported (Jiang et al. 2012; Liu et al. 2012; Jiang et al. 2015; Ni et al. 2015; Tian et al. 2016), but reports of other cloned blast genes in rice were rare. Here, we introduced 9 cloned blast resistance genes-*Pi37*, *Pit*, *Pid3*, *Pigm*, *Pi36*, *Pi5*, *Pi54*, *Pikm* and *Pb1-*into 4 rice varieties and evaluated their applications in breeding.

In our study, the blast resistance of 9 donor parents was evaluated under natural infection conditions in 2014, 2015 and 2016. Four donor parents—Gumei4 (*Pigm*), Digu (*Pid3*), Tetep (*Pi54*) and Q1333 (*Pi37*)—have high resistance to leaf and neck blast, four donor parents K59 (*Pit*), Tsuyuake (*Pikm*), Modan (*Pb1*) and RIL260 (*Pi5*) have medium or high resistance to leaf blast and susceptible to neck blast, while Q61 (*Pi36*) was susceptible to leaf and neck blast. From the results, we can see that the resistance of donor parents could differ even under the same infection conditions.

We used BC_1_F_2_ and BC_2_F_2_ populations to evaluate the effects of individual genes in different backgrounds. Additive and dominant effects were used to evaluate gene effects in F_2_ population. Gene effects differed in the four rice backgrounds. The gene *Pigm* and *Pid3* showed significantly enhanced resistance to blast in all four rice backgrounds, but the gene *Pb1* showed no significant difference in rice blast resistance than the control. The same gene might also perform differently in different years in the same background. The blast resistance of gene *Pi54* was better in 2015 than in 2014 in the background of Y58S. The reason for this is mainly due to the different gene effects influenced by different climate or physiological factors.

From the blast resistance performance of the 9 genes in 3 years in Xianfeng, we can see that *Pid3* and *Pigm* have better resistance than the other genes, so these two genes are valuable for application in the Wuling mountain area. *Pigm* have a broad-spectrum and durable resistance to rice blast that confers durable resistance to the fungus *Magnaporthe oryzae* without yield penalty through epigenetic regulation of paired antagonistic NBS-LRR (Deng et al. 2017). The genes *Pi54*, *Pi37* and *Pi5* should be used according to background. Genes such as *Pb1* and *Pit* have little resistance to blast, mainly because the fungus *Magnaporthe oryzae* in Xianfeng can overcome them, so these genes are not suitable for the Xianfeng area, although they may be suitable in other areas.

Two blast resistance genes *Pi54* and *Pikm* on rice chromosome 11 were used to improve blast resistance of the sterile lines through MAS with different markers with the distance of ∼2.5 Mb. Genes *Pikm*, *Pik-h* and *Pik-p* were mapped on the *Pik* locus, and these genes comprised a pair of NBS-LRR genes, but gene *Pi54* encodes one NBS-LRR protein. The genes *Pi54*, *Pikm*, *Pik-h* and *Pik-p* were clone from rice variety Tetep, Tsuyuauke, K3 and K60, respectively. Donor parents Tetep and Tsuyuauke were high or medium resistant to rice blast in three years, and K3 and K60 may have a potentially value in rice blast resistance breeding.

Broad spectrum and durable resistance are the major objectives of rice blast resistance breeding. Most efforts in breeding for blast resistance have been directed towards incorporating single genes. Rice varieties containing only one major resistance gene have a tendency to break down as unpredictable changes occur in the composition of pathogen populations, so pyramiding more blast resistance genes in a rice cultivar may solve this problem (Ahn and Ou 1982; Kiyosawa 1982). Gene pyramiding can overlap different resistance genes, which seems promisingly to provide broad spectrum and durable resistance (Tabien et al. 2002). In this study, we developed lines containing two blast resistance genes Y58S (*Pi37*/*Pid3*), GZ63S (*Pi5*/*Pi54*), GZ63S (*Pi54*/*Pid3*) and C815S (*Pigm*/*Pid37*) that were highly resistant to leaf and neck blast under natural infection conditions, and the phenotypes showed that the effect on neck blast was better than on leaf blast in the Y58S, GZ63S and C815S backgrounds.

Most rice varieties lose their resistance after a few years planted in the same area because many varieties of single resistance genes must cope with new *M. oryzae races*. Studies have indicated that the genetic control of blast resistance is complex and involves both major and minor resistance genes with complementary or additive effects, as well as environmental interactions. Mapping new blast resistance genes and developing durable resistance varieties are, therefore, of high value. To pursue durable resistance to blast, we should exploit genetic diversity, which is an ecological approach to disease control that can be highly effective over a large area and contribute to the sustainability of crop production (Zhu et al. 2000). In this study, we introduced 9 cloned blast resistance genes into 4 recurrent parents. The effects of the genes on blast resistance were evaluated in the natural environment, and the results provide an important theoretical basis for the utilization of these rice blast resistance genes in China.

## Additional file

Additional file 1: **Table S1.** SSR or InDel markers used for selection of blast resistance genes. (DOCX 19 kb)
