# Supplementary material for: Development and evaluation of improved lines with broad-spectrum resistance to rice blast using nine resistance genes
Source: Rice (N Y). 2019 May 6;12:29. doi: 10.1186/s12284-019-0292-z (PMC6502921; doi:10.1186/s12284-019-0292-z)
Supplement: Supplementary file 1 — Table S1. SSR or InDel markers used for selection of blast resistance genes. (DOCX 19 kb) [file 12284_2019_292_MOESM1_ESM.docx]

**Table S1** SSR or InDel markers used for selection of blast resistance genes

| Gene | Marker | Marker type | Chr | Distance | Forward primer (5'-3') | Reverse primer (5'-3') |
| --- | --- | --- | --- | --- | --- | --- |
| *Pit* | RM10125 | SSR | 1 | 2376851 | CTGTACAAGAACGGCAGCAACC | GCACCACCAAACAGAGACAGAGG |
|  | LJT-3** | InDel | 1 | 2692489 | GAGAATATCTTATGACAAACTAG | CATCTACGTTCTAATCCCCCAACG |
| *Pi37* | RM11726 | SSR | 1 | 32542477 | CCAATCCTTTCCAAATCCAACG | CCAAATATCTAGCCTCCTATTGAGC |
|  | LJ-1** | InDel | 1 | 33144588 | CATGATGGTTCTCACCTCACA | CGCTAGAGAGTGCAACGAAA |
| *Pigm* | Pi2-4* | InDel | 6 | 10365923 | CGGTAAGAGTAACACCAAGC | GACGTGCGAGTTGTGACAGCT |
|  | HC26* | SSR | 6 | 10468548 | GACCCAACAAGTCCAGCACTA | CATGCTCTCCCAGATCAAGAA |
|  | HC3* | SSR | 6 | 10121927 | ACCATCTTGTGCGACCCTAC | TCTCAGCACACAAGAGTTTACCA |
| *Pid3* | LJ3-1** | InDel | 6 | 12990572 | GCTCCTAGTGTGTTAGCTCTGCG | GCGCATCCATGCACATAATACAAC |
|  | RM19951 | SSR | 6 | 12984054 | TCCCTTAGGTAGTCAGGTCACAGC | GTCTATGTCGTCAGTATCGTTGATGG |
| *Pi36* | RM22385 | SSR | 8 | 2945117 | CCTCCAGCTTCTGTCTCCTTCACTCC | GGAAGTGTAATGCTCATGCCTTTGC |
|  | LJ-2** | InDel | 8 | 2854057 | GGTCGTGTGGGCTCCTATC | TATGTTGGGACGGAGGAAAG |
| *Pi5* | RM24019 | SSR | 9 | 9593599 | GGACAAGATGATGACACTCTGTTACC | TGCTTCTTAGTTCTCAGCCACACC |
|  | LJ-7** | InDel | 9 | 9664652 | CCAAATCAGGGAAATCAAGC | GGATTACCTCCAGACGCAGT |
|  | RM24034 | SSR | 9 | 9869084 | CATCGATATGGTGCAGAGAATGC | GCAGGCAAAGATCTCCAAGTAGC |
| *Pi54* | RM27150 | SSR | 11 | 24324517 | ATTCAGGCTCGCTTACCATCTCC | CCTCTGCTTGTCCCAAATCACC |
|  | RM27181 | SSR | 11 | 24924151 | CAATTCAGAGGAGCAAGGTGTCC | TTCTTAACCTGGACTTGCCATGC |
|  | RM27189 | SSR | 11 | 24984877 | CCGAGCTTAATTTGCATCTACTGC | TGCAGATTGTGGTTGGAAATGG |
| *Pb1* | RM26998 | SSR | 11 | 21777074 | ACGCACGCACATCCTCTTCC | CGGTTCTCCATCTGAAATCCCTAGC |
|  | RM26964 | SSR | 11 | 21043268 | GGGCGTTTCTCTCTTCTTCAGG | CCGAATATAAAGAGAGGAAGGTGAGG |
| *Pikm* | RM224 | SSR | 11 | 27673353 | ATCGATCGATCTTCACGAGG | TGCTATAAAAGGCATTCGGG |
|  | LJM-1* | InDel | 11 | 27828537 | GCCTAAGTTACCAAATGG | TATACACAAGGTCTCCTTTC |

Closely-linked or cosegregative markers with *, functional markers with **.
